# Pneumonia in hospitalized neurologic patients: trends in pathogen distribution and antibiotic susceptibility

**DOI:** 10.1186/s13756-019-0475-9

**Published:** 2019-02-01

**Authors:** Han Sang Lee, Jangsup Moon, Hye-Rim Shin, Seon Jae Ahn, Tae-Joon Kim, Jin-Sun Jun, Soon-Tae Lee, Keun-Hwa Jung, Kyung-Il Park, Ki-Young Jung, Manho Kim, Sang Kun Lee, Kon Chu

**Affiliations:** 1 Department of Neurology, Laboratory for Neurotherapeutics, Biomedical Research Institute, Seoul National University Hospital, 101 Daehak-ro, Jongno-gu, Seoul, 110-744 South Korea; 2 Department of Neurology, Ajou University School of Medicine, Suwon, South Korea; 3 Department of Neurology, School of Medicine, Kyungpook National University, Kyungpook National University Chilgok Hospital, Daegu, South Korea; 4 Department of Neurology, Seoul National University Healthcare System Gangnam Center, Seoul, South Korea; 5 Department of Neurosurgery, Seoul National University Hospital, Seoul, South Korea

**Keywords:** Nosocomial infection, Pneumonia, Antimicrobial resistance

## Abstract

**Background:**

Bed-ridden state, dysphagia, altered mental state, or respiratory muscle weakness are common in neurologic patients and increase the risk of pneumonia. The major causes of pneumonia in neurologic patients may differ from those in the general population, resulting in a different pathogen distribution. We investigated the trends of pathogen distribution in culture-positive pneumonia in hospitalized neurologic patients and the related antibiotic resistance in those with hospital-acquired pneumonia (HAP).

**Methods:**

A retrospective study was performed at Seoul National University Hospital, South Korea. Patients admitted to the Department of Neurology with a positive respiratory specimen culture between 2007 and 2016 were included. Pneumonia events in patients were screened by chronologically associating the date of respiratory specimen acquisition for culture studies and the date of antibiotics administration. Subgroup analyses regarding multidrug resistance in HAP were performed in different pneumonia categories, by presence of ≥1 risk factor and by time period (first half vs. second half of study period). Microbial resistance profiles of isolates from patients with pneumonia were analyzed.

**Results:**

We identified 351 pneumonia cases in 227 patients involving 36 different pathogens. 232 cases were HAP, of which 70 cases were intensive care unit (ICU)-HAP. The leading pathogens were *Stapylococcus aureus*, *Klebsiella pneumoniae*, *Acinetobacter baumannii*, *Pseudomonas aeruginosa*, *Streptococcus pneumoniae,* and *Enterobacter aerogenes,* which were isolated in 133 (37.9%), 72 (20.5%), 55 (15.7%), 44 (12.5%), 33 (9.4%), and 27 (7.7%) cases, respectively. Cases with HAP showed a higher proportion of *P. aeruginosa* and a lower proportion of *S. pneumoniae* (both, *p* < 0.05) than those with non-HAP. ICU-HAP isolates showed a higher multidrug resistance (MDR) rate than non-ICU-HAP isolates (*p* < 0.005) in those with ≥1 MDR risk factor. Non-susceptibility to imipenem (*p* < 0.0005), piperacillin-tazobactam (*p* < 0.001), cefepime (*p* < 0.005), and trimethoprim-sulfamethoxazole (*p* < 0.05) in Gram-negative pathogens increased over time in both ICU and non-ICU settings.

**Conclusions:**

*S. aureus*, *K. pneumoniae*, *A. baumannii*, *P. aeruginosa*, *S. pneumoniae,* and *E. aerogenes* were the leading isolates in culture-positive pneumonia in hospitalized neurologic patients. Antimicrobial resistance of Gram-negative pathogens in neurologic patients with culture-positive HAP has recently increased.

**Electronic supplementary material:**

The online version of this article (10.1186/s13756-019-0475-9) contains supplementary material, which is available to authorized users.

## Background

Nosocomial infection results in higher mortality, an extended hospitalization period, and escalated healthcare costs [1–3]. Nosocomial infection, especially urinary tract infection and pneumonia, has been estimated to develop in one-third of stroke patients, who comprise the majority of neurologic patients [4, 5]. Hospitalized patients with neurologic diseases are especially prone to pneumonia, in which a bed-ridden state, dysphagia, an altered mental state, or mechanical ventilation due to respiratory muscle weakness can all increase its risk [6].

Early empiric coverage for possible pathogens of hospital-acquired pneumonia (HAP) is crucial to improve the prognosis, which requires knowledge of recent prevalence of antibiotic-resistant pathogens such as methicillin-resistant *Staphylococcus aureus* and multidrug-resistant nonfermenters [7, 8]. The distribution of pathogens causing HAP and their antibiotic resistance profiles differ according to region and individual circumstances (e.g. intensive care unit (ICU) setting vs. non-ICU setting, local microbial flora) [8–10]. Treatment guidelines have been proposed in both Western and Asian countries [10, 11]. However, neurologic patients comprise only a limited number within most data. Therefore, we investigated trends in microbial etiology of pneumonia in hospitalized patients with neurologic diseases, along with changes in the antibiotic resistance profiles of common pathogens.

## Methods

### Patient screening and inclusion

Data were retrospectively reviewed via the electronic medical record (EMR) system of Seoul National University Hospital (SNUH), South Korea. Data of patients who were admitted to the Department of Neurology from January 1, 2007 to December 31, 2016 were collected.

Patients with at least one positive respiratory specimen culture and/or blood culture result were included. Respiratory specimens with a Grams-stain result showing ≥25 squamous epithelial cells per low-power field were considered as inadequate samples and were excluded. Respiratory specimens were collected using upper respiratory tract suction, transtracheal aspiration (through the tracheostomy site), endotracheal aspiration (through the endotracheal tube), bronchoalveolar lavage, or as expectorated sputum.

To screen events of infectious diseases, we selected respiratory cultures chronologically related to the administration of antibiotic agents. Specifically, those with a history of at least 4 days of antibiotic administration and those whose antibiotics were administrated within 3 days of the first blood and/or respiratory specimen culture were included. Patients with a history of antibiotic administration ≤3 days were excluded because short antibiotic administration periods were mostly observed in those with causes other than pneumonia, such as aspiration pneumonitis. Patient data were anonymized to protect patient privacy.

### Definitions

**Fig. 1:**
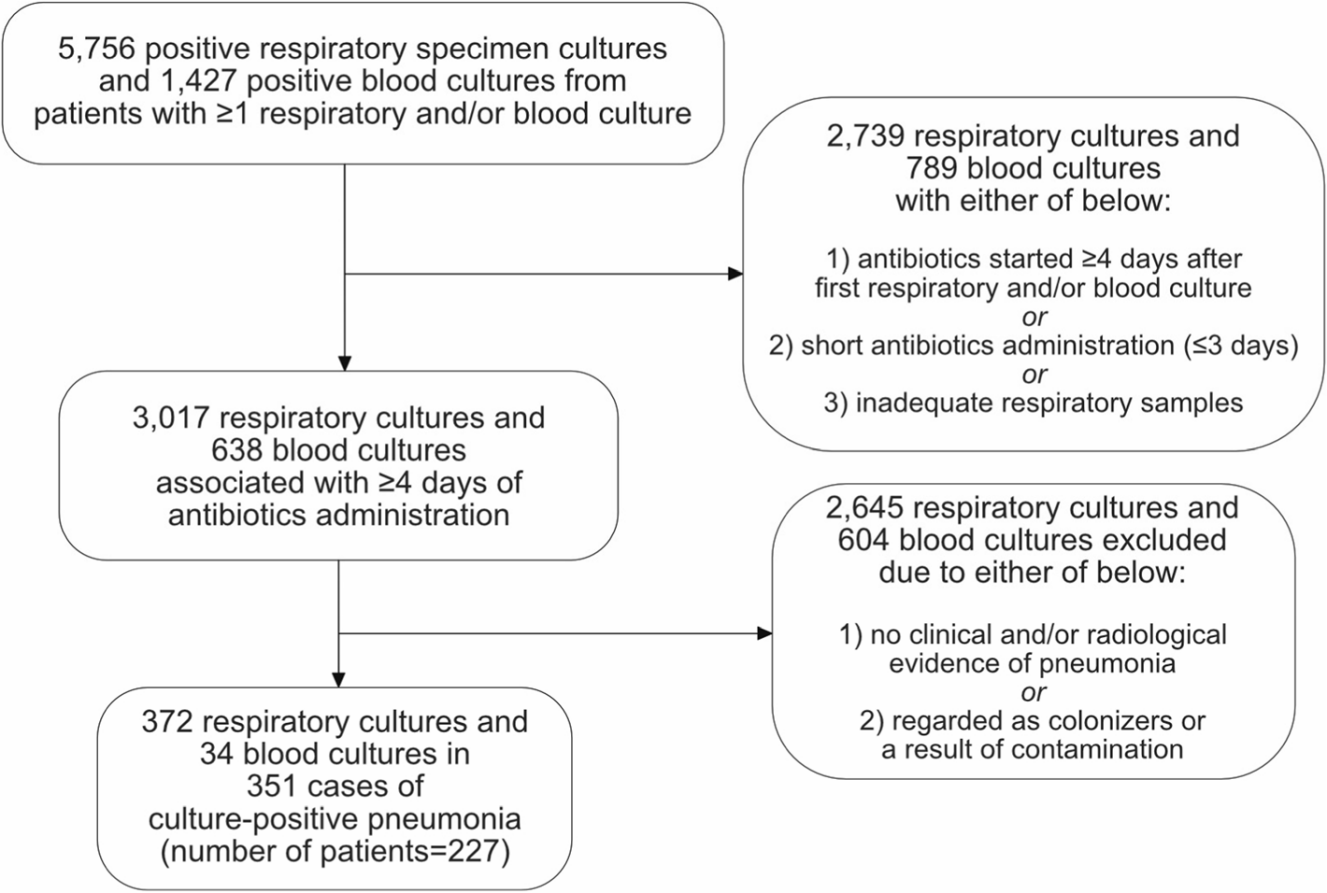
Patient selection flow chart. Of 5756 and 1427 positive respiratory specimen cultures and blood cultures, respectively, 372 respiratory cultures and 34 blood cultures involving 227 patients and 351 pneumonia cases were finally included

Pneumonia was defined as fever and/or leukocytosis with either clinical (rales or new onset of purulent sputum) or radiological (new or progressive infiltrate, consolidation, cavitation, or pleural effusion) evidence of pneumonia. EMR was reviewed to exclude patients without evidence of pneumonia. Clinically or radiologically ambiguous cases were only included if the primary physician and a consulted expert in infectious diseases both agreed upon the case as pneumonia. Cultured microorganisms were considered as pathogens if a culture was obtained under the diagnosis of pneumonia, but were considered as colonizers or a contamination if the patient clinically improved without an adequate microbial therapy (Fig. 1).

Cases of pneumonia were classified by the period of occurrence (early or late), the location of infection (community-acquired, health-care-associated, or hospital-acquired) with hospital-acquired pneumonia further categorized to ICU-related or non-related, multi-drug resistance (MDR) of the pathogen, and the presence of any risk factor of multi-drug resistant pathogen infection in hospital-acquired pneumonia.

The first half of the study period (from January 1, 2007 to December 31, 2011) was defined as the early period and the second half of the study period (from January 1, 2012 to December 31, 2016) was defined as the late period.

Hospital acquired-pneumonia was defined as an onset of pneumonia more than 48 h after admission [10, 12]. HAP was further subclassified into intensive care unit (ICU)-HAP or non-ICU-HAP depending on the location of the patient at the onset of pneumonia. Patients with HAP were classified as ICU-HAP if the first signs of pneumonia were noted after 48 h in intensive care. Pneumonia occurring in the first 48 h after ICU discharge were also categorized as ICU-HAP. Otherwise, the patients were classified as non-ICU-HAP. Cases were defined as healthcare-associated pneumonia (HCAP) if the onset of pneumonia preceded or was within 48 h of admission and the patient had a separate history of hospitalization ≥2 days (either at SNUH or at other hospitals) within the previous 90 days, or had residence in a nursing home or extended care facility [12]. Otherwise, cases were defined as community-acquired pneumonia (CAP).

MDR was defined for the most common pathogens of pneumonia from our data. We followed definitions proposed by the European Centre for Disease Prevention and Control for defining MDR of *S. aureus*, *Klebsiella pneumoniae* (*K. pneumoniae*), *Acinetobacter baumannii* (*A. baumannii), Pseudomonas aeruginosa* (*P. aeruginosa*), and *Enterobacter aerogenes* (*E. aerogenes*) [13]. In short, non-susceptibility to ≥1 agent in ≥3 antimicrobial categories was defined as MDR. *S. aureus* was also defined as multidrug resistant if identified as methicillin-resistant. MDR of *Streptococcus pneumoniae* (*S. pneumoniae*) was defined as resistance to ≥2 classes of the following antimicrobial agents: penicillin, ceftriaxone, erythromycin, levofloxacin, tetracycline, and trimethoprim-sulfamethoxazole [14]. Antibiotic susceptibility results were reported as qualitative results (i.e. resistant, intermediate, or susceptible) with/without quantitative results (minimum inhibitory concentration). All susceptibility results were tested using the guidelines of Clinical & Laboratory Standards Institute (CLSI).

Risk factors for MDR pathogens in HAP were estimated following the American Thoracic Society (2005) guidelines [12]. Specifically, late onset (≥5 days after current admission), antimicrobial therapy in the preceding 90 days, hospitalization ≥2 days in the preceding 90 days, residence in a nursing home or an extended care facility were regarded as risk factors for MDR.

### Statistical analysis

A Fisher’s exact test was used to compare categorical variables. Proportional changes in individual antibiotic susceptibilities or MDR of common Gram-negative bacteria throughout the study periods were compared using the Cochran–Mantel–Haenszel test. All probabilities were two-tailed and a *p*-value of less than 0.05 was considered to be statistically significant. R (version 3.4.4) was used to perform the analysis.

## Results

### Frequency of each pathogen

**Fig. 2:**
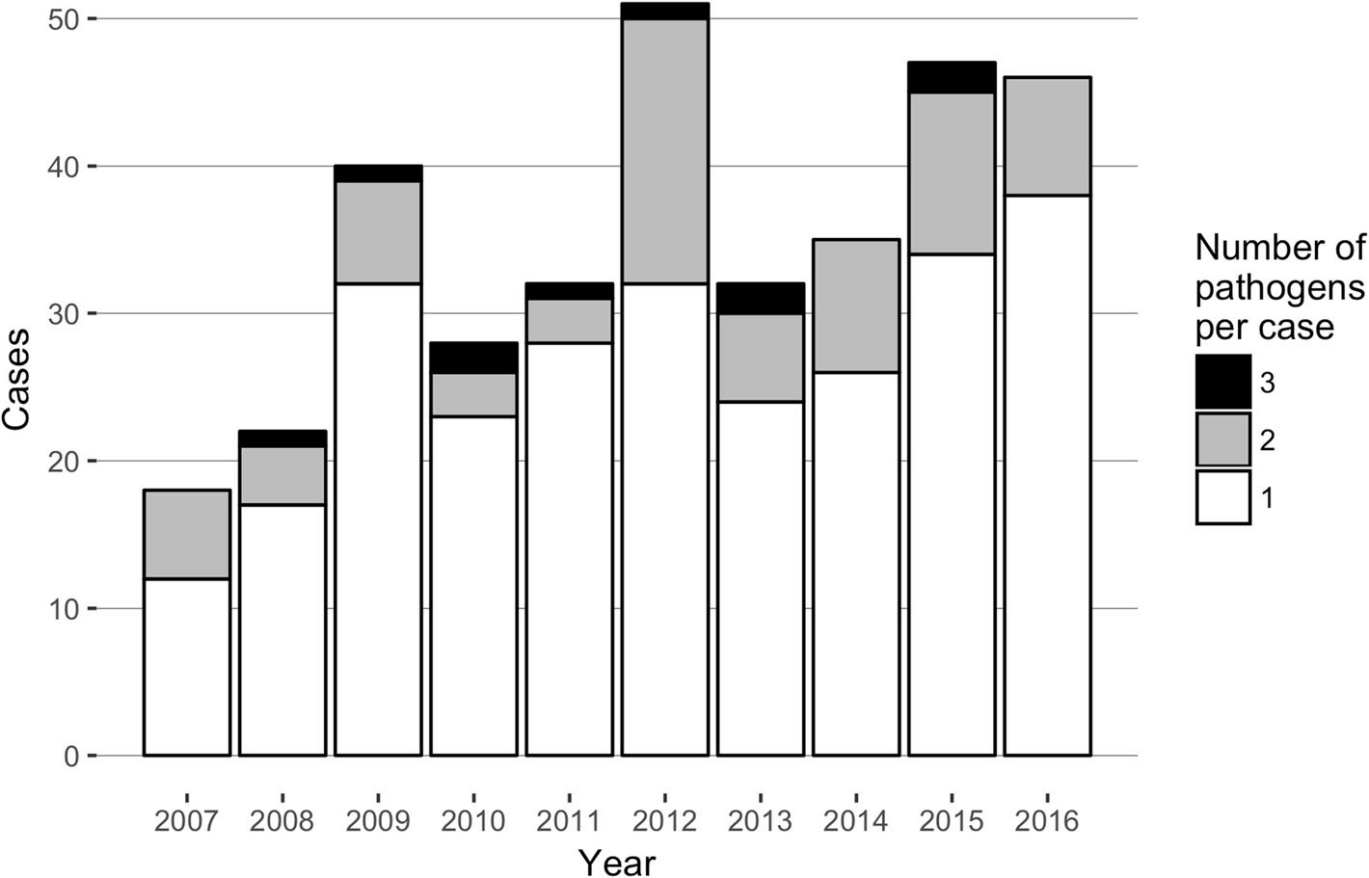
Number of culture-positive pneumonia cases and isolated pathogens per year. Eighty-five cases (24%) had multiple pathogens (75 cases with 2 pathogens and 10 cases with 3 pathogens)

Of 5756 positive respiratory specimen culture results and 1427 positive blood culture results involving 1417 patients (F:M = 564:853), 3071 respiratory specimen culture results and 638 blood culture results from 996 patients were associated with a ≥ 4 days antibiotics-administration. After reviewing the EMR and radiological data, 351 pneumonia cases involving 227 patients (age, 62.9 ± 18.9 years; F:M = 81:196) were finally included. Eighty-five cases (24%) had multiple pathogens (75 cases with 2 pathogens and 10 cases with 3 pathogens) (Fig. 2). The majority of cases were HAP (70 and 162 ICU- and non-ICU-HAP, respectively), with 65 HCAP cases and 54 CAP cases.

**Fig. 3:**
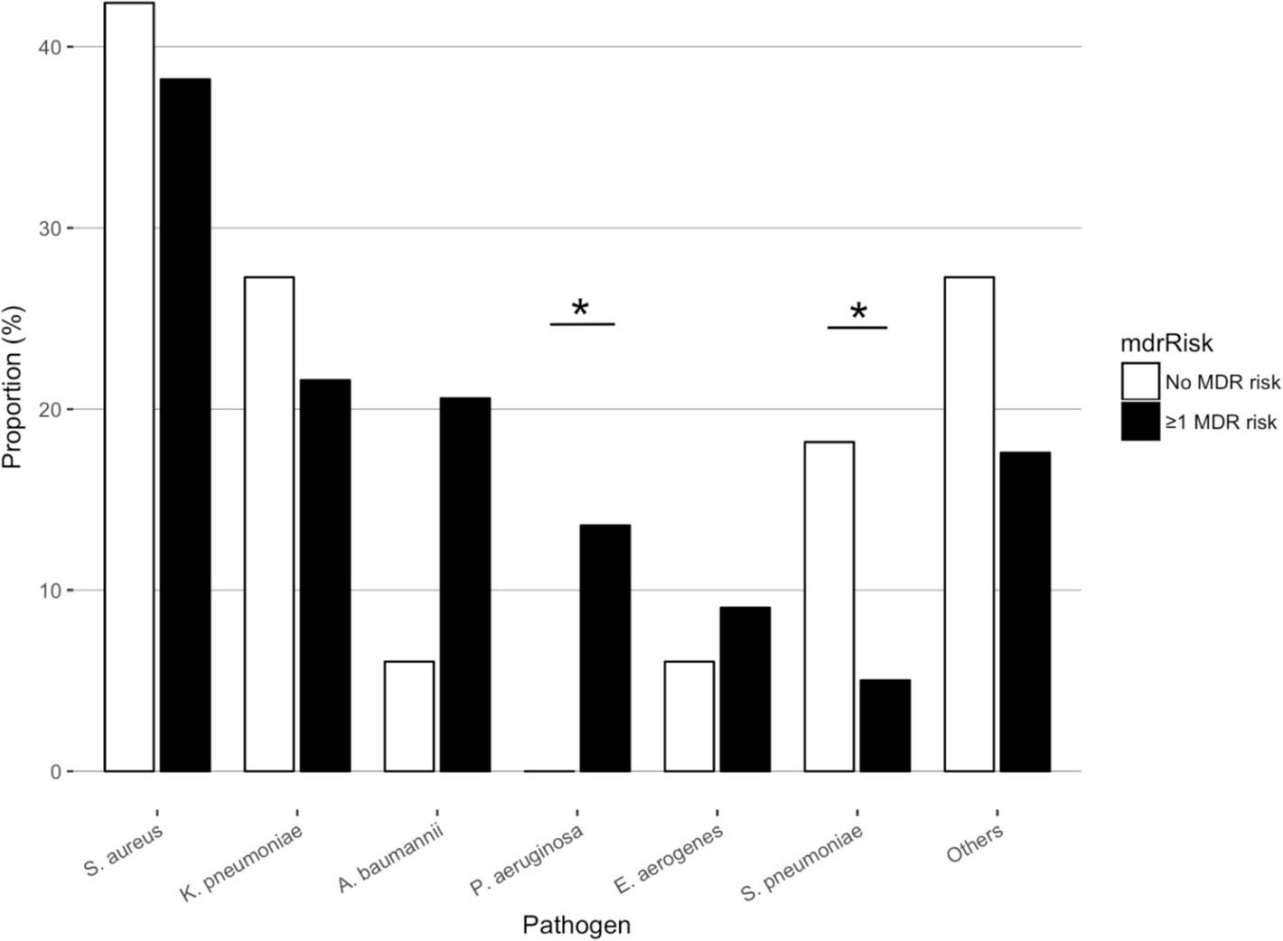
Proportion of each pathogen in HAP cases with or without MDR risk factors. The frequency of each pathogen differed by whether each case had a risk factor for MDR. In those with ≥1 risk factor, *P. aeruginosa* were more frequent and *S. pneumoniae* was less frequent. *p < 0.05

We identified 36 different pathogens. The most frequent pathogens in total were *S. aureus*, *K. pneumoniae*, *A. baumannii*, *P. aeruginosa*, *S. pneumoniae,* and *E. aerogenes,* which were isolated in 133 (37.9%), 72 (20.5%), 55 (15.7%), 44 (12.5%), 33 (9.4%), and 27 (7.7%) cases, respectively (Table 1, Additional file 1: Figures S1 and S2). There was an overall increment in the proportion of non-ICU-HAP cases. In the 232 cases of HAP, *S. aureus* (38.8%), *K. pneumoniae* (22.4%), *A. baumannii* (18.5%), *P. aeruginosa* (11.6%), *E. aerogenes* (8.6%), and *S. pneumoniae* (6.9%) were the most frequent pathogens. *A. baumannii* was more frequent and *S. pneumoniae* was less frequent in HAP cases than in non-HAP cases (both, *p* < 0.05). Moreover, among the HAPs, the frequency of each pathogen differed according to whether a patient had a risk factor for MDR. In 199 patients with ≥1 risk factor, *S. aureus* (38.2%), *K. pneumoniae* (21.6%), *A. baumannii* (20.6%), and *P. aeruginosa* (13.6%) were the leading isolates, while in 33 patients without a risk factor, *S. aureus* (42.4%), *K. pneumoniae* (27.3%), *S. pneumoniae* (18.2%), and *A. baumannii* (6.1%) were the leading isolates (Fig. 3).

**Table 1:** Frequency of isolated pathogens in HAP and non-HAP pneumonia cases from 2007 to 2016

	HAP		Non-HAP	Total
	ICU	Non-ICU		
*S. aureus*	32 (45.7%)	58 (35.8%)	43 (36.1%)	133 (37.9%)
*K. pneumoniae*	9 (12.9%)	43 (26.5%)	20 (16.8%)	72 (20.5%)
A. baumannii*	22 (31.4%)	21 (13.0%)	12 (10.1%)	55 (15.7%)
*P. aeruginosa*	3 (4.3%)	24 (14.8%)	17 (14.3%)	44 (12.5%)
S. pneumoniae*	1 (1.4%)	15 (9.3%)	17 (14.3%)	33 (9.4%)
*E. aerogenes*	9 (12.9%)	11 (6.8%)	7 (5.9%)	27 (7.7%)
Others	10 (14.3%)	34 (21.0%)	38 (31.9%)	82 (23.4%)
total	70 (100%)	162 (100%)	119 (100%)	351 (100%)

*A. baumannii* was more frequent and *S. pneumoniae* was less frequent in HAP cases than in non-HAP cases. *p < 0.05

### MDR in HAP cases

MDR was more prevalent in HAP cases with ≥1 MDR risk factor, among which ICU-HAP cases more frequently showed MDR than non-ICU-HAP cases. In 265 isolates from 204 HAP cases involving the 6 most frequent pathogens, the frequency of MDR was evaluated (Additional file 1: Table S1). Isolates from cases with at least one MDR risk factor showed a significantly higher rate of MDR compared to those from cases without any MDR risk factor (*p* < 0.001). Specifically, 7 (19.4%) out of 36 isolates from 26 cases without any MDR risk factor showed MDR, while 141 (61.6%) out of 229 isolates from 178 cases with ≥1 MDR risk factor showed MDR. MDR was seen in 58 (75.3%) of 77 isolates from ICU-HAP cases with ≥1 MDR risk factor, while 83 (54.6%) of 152 isolates from non-ICU-HAP cases with ≥1 MDR risk factor showed MDR (*p* < 0.005).

The proportion of MDR of common Gram-positive bacteria (i.e. *S. aureus* and *S. pneumoniae*) was compared between the early and late years of the study period. *S. aureus* showed MDR in 67 of the 90 cases of HAP with *S. aureus* infection (Additional file 1: Table S2). However, there was no statistically significant change in the frequency of multidrug-resistant *S. aureus* in the early and late study periods (*p* = 0.834). Thirteen (86.7%) of 15 cases and 14 (63.6%) of 22 cases were due to multidrug-resistant *S. aureus* in the early years in the ICU and non-ICU settings, respectively, while 15 (88.2%) of 17 cases and 25 (69.4%) of 36 cases in the late years showed MDR, respectively. All multidrug-resistant *S. aureus* isolates showed resistance to oxacillin. Only one case, which occurred in 2015 from a non-ICU setting, showed an intermediate susceptibility result to vancomycin. Of the 16 HAP cases (6 in the early periods and 10 in the late periods) due to *S. pneumoniae*, 50% of cases were due to multidrug-resistant *S. pneumoniae* in each period.

The most common Gram-negative bacteria in HAP were *K. pneumoniae*, *A. baumannii*, *P. aeruginosa*, and *E. aerogenes*. The number of HAP events due to the common Gram-negative bacteria in the early and late periods were 47 and 84, respectively. The frequency of MDR of these pathogens did not significantly differ between the early and late study periods (Table 2).

**Table 2:** Frequency of MDR and non-MDR Gram-negative pathogens in the early and late periods of the study

	Study period	Number of MDR cases	Number of non-MDR cases	*p*-value
*A. baumannii*	Early	7	2	1
	Late	28	6	
*K. pneumoniae*	Early	7	9	1
	Late	15	21	
*P. aeruginosa*	Early	1	13	0.076
	Late	5	8	
*E. aerogenes*	Early	1	8	1
	Late	1	10	

### Trends in antibiotic susceptibility of gram-negative bacteria

**Fig. 4:**
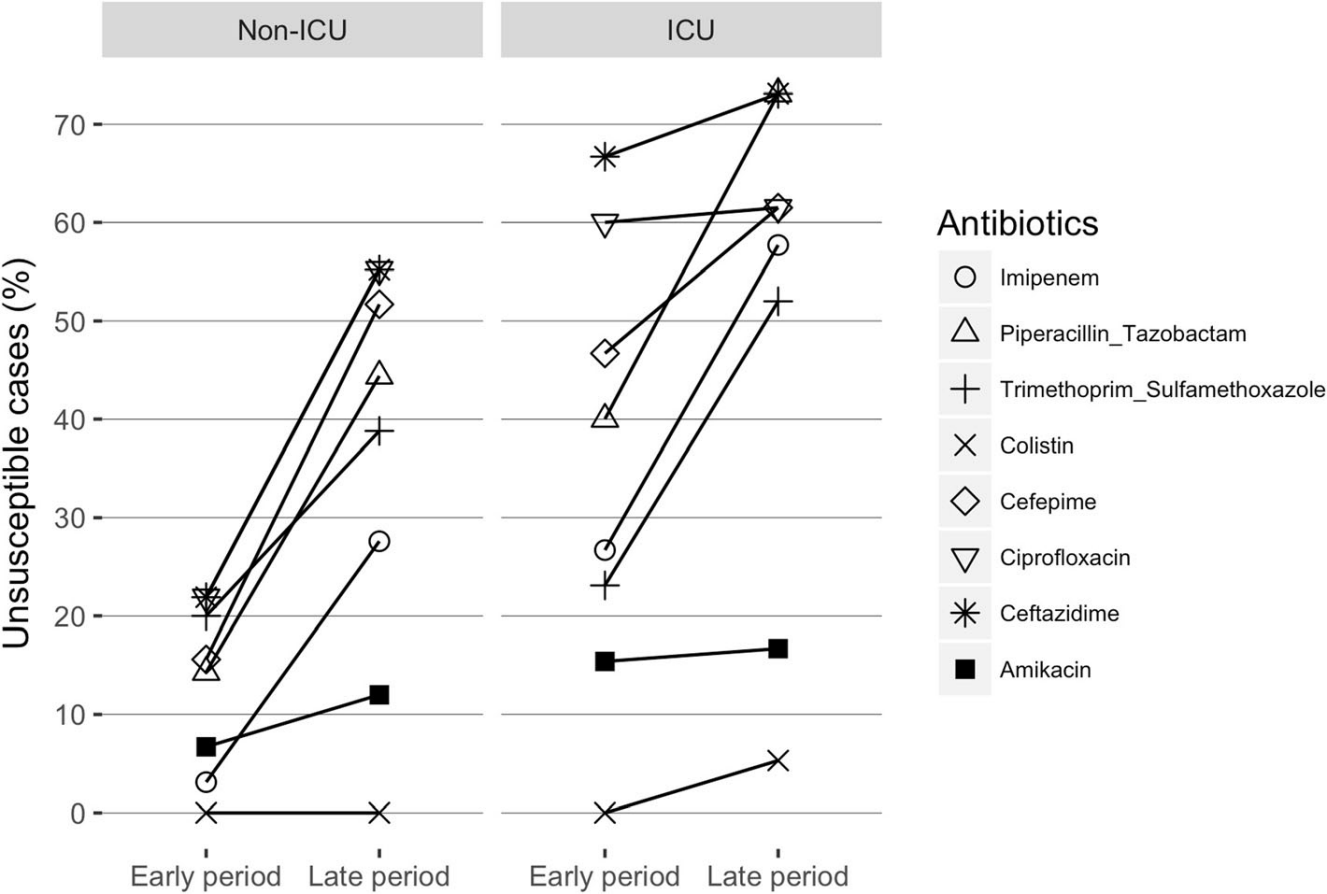
Trends of susceptibility to each antibiotic agent in the early (2007–2011) and late (2012–2016) periods of the study. Non-susceptibility to imipenem, piperacillin-tazobactam, cefepime, and trimethoprim-sulfamethoxazole increased in both ICU and non-ICU settings, while non-susceptibility to ciprofloxacin and ceftazidime increased in non-ICU settings but remained high in ICU settings. *p < 0.05, **p < 0.01, ***p < 0.005, ****p < 0.0001

When we compared the resistance to each antibiotic agent of the common Gram-negative bacteria in the early and late study periods, an overall increasing trend in the proportion of cases with pathogens showing non-susceptibility (i.e. resistant or intermediate results) to each antibiotic agent was seen (Fig. 4). Antibiotic susceptibilities to ciprofloxacin, ceftazidime, cefepime, and imipenem were tested in all 131 cases while susceptibilities to piperacillin-tazobactam, amikacin, trimethoprim-sulfamethoxazole, and colistin were tested in 123, 111, 107, and 64 cases, respectively. Increasing non-susceptibility to various antibiotic agents was seen in both the ICU and non-ICU settings. Specifically, non-susceptibility to imipenem (*p* < 0.0005), piperacillin-tazobactam (*p* < 0.001), cefepime (*p* < 0.005), and trimethoprim-sulfamethoxazole (*p* < 0.05) increased in both the ICU and non-ICU settings, while non-susceptibility to ciprofloxacin (*p* < 0.05) and ceftazidime (*p* < 0.01) increased in non-ICU settings but remained high in ICU settings. The proportion of non-susceptibility to amikacin (6.7, 12.0, 15.4, and 16.7% in the non-ICU early period, non-ICU late period, ICU early period, and ICU late period, respectively) and colistin (0% in non-ICU setting of both periods and ICU early period, and 5.3% in the ICU late period) remained low throughout the years in both settings. Trends in antibiotic resistance to common Gram-positive bacteria, *S. aureus* and *S. pneumoniae*, were separately evaluated because the types of antimicrobial susceptibility tests performed were different in each bacteria (Additional file 1: Figures S2-S4).

## Discussion

We analyzed the distribution of pathogens of hospitalized patients with culture-positive pneumonia, pathogen changes in relation to MDR, and the susceptibility to individual antibiotic agents in a single center. The most frequent pathogens were *S. aureus*, followed by *K. pneumoniae*, *A. baumannii*, *P. aeruginosa*, and *E. aerogenes*. *P. aeruginosa* was more frequent *and S. pneumoniae* was less frequent in HAP cases than in non-HAP cases. In the presence of at least one MDR risk factor, MDR was more frequent in ICU settings than in non-ICU settings. Although there was no significant change in the frequency of MDR in any pathogen throughout the study period, non-susceptibility to individual antimicrobial agents in Gram-negative bacteria significantly increased.

Compared to previous studies, our study showed a higher proportion of *K. pneumoniae* in isolates from culture-positive HAP. A study from the SENTRY program showed that, in the United States and Europe, *S. aureus* (range, 23.0–36.2%) and *P. aeruginosa* (range, 19.7–20.8%) were the predominant isolates in HAP while *K. pneumoniae* and *A. baumannii* accounted for a lower proportion (range, 8.5–10.1% and range, 4.8 to 5.6%, respectively) [15]. Another prospective surveillance study in Asian countries showed *S. aureus* was the predominant pathogen in HAP in South Korea and Singapore (range, 26.7–30.7%), while *K. pneumoniae* (range, 25.0–30.6%), *A. baumannii* (range, 16.2–27.2%), and *P. aeruginosa* (range, 10.0–21.9%) were the predominant pathogens in other countries [8]. Interestingly, a recently published systematic review of pneumonia complicating stroke showed a high proportion (21.8%) of *K. pneumoniae* from culture-positive pneumonia [16].

Although the proportion of MDR in each Gram-negative pathogen did not significantly increase throughout the study period, the proportion of non-susceptibility to several antibiotic agents in Gram-negative pathogens were significantly increased. In our study, there was an increase in non-susceptibility to imipenem, piperacillin-tazobactam, cefepime, ciprofloxacin, and ceftazidime, which are antibiotics targeting Gram-negative bacteria in HAP with MDR risk factors, suggesting low antimicrobial effects in cases where these antibiotics are empirically administered. Numerous studies have shown a recent increasing trend in antimicrobial resistance in Gram-negative bacteria [17–20]. Data from the Surveillance Network showed non-susceptibility to carbapenems in *A. baumannii* increased from 22 to 52% from 2002 to 2008. Similarly, non-susceptibility to carbapenems in *A. baumannii* in our study increased from 55.6 to 83.8%, which is strikingly high compared to studies published in recent years [8, 15, 21].

Our study consisted of data across a 10-year study period involving clinical and/or radiologically defined cases of pneumonia. The response to antibiotics was considered when making etiologic diagnoses so as not to mistake commensal bacteria as pathogens. Our study shows a definite trend of increasing resistance to commonly used antibiotics in Gram-negative pathogens, and a higher prevalence of MDR pathogens in HAP in ICU settings, and in patients with MDR risk factors.

Our study has some limitations. The cases included in our study consisted of culture-positive pneumonias only. A study comparing the outcome of ICU-acquired pneumonia showed negative microbiologic findings were associated with a shorter duration of antimicrobial treatment and better survival [22]. Since our study did not include culture-negative pneumonias, it may show results with a higher proportion of pathogens with MDR or higher virulence. The screening method using the number of days of antibiotic administration to detect a pneumonia event may also have impact on the results. Although most events with a short antibiotic administration period would be those without a definite evidence of infection such as aspiration pneumonitis, there is also a possibility of short administration due to early mortality. This may have resulted in excluding critically-ill patients close (within 3 days) to death from our study. Overall, our study excluded culture-negative pneumonia, which is common in clinical practice, and may have also excluded cases with positive culture results due to the limitations of our screening methods. Regarding this study was done in culture positive pneumonia patients who have had antibiotic therapy for 4 days or more, extrapolating the results to other populations is hampered, and they cannot be used to guide empirical therapy in the days before the culture result is known. Changes in the MIC breakpoints in the CLSI guidelines should be accounted for the rise of antimicrobial resistance to some antibiotics. Recently, CLSI lowered the breakpoints for several beta-lactam antibiotics, potentially leading more isolates reported as intermediate or resistant to the tested antibiotics [23, 24]. Our study was performed in a single neurologic center, which, considering that hospital personnel may act as a vehicle of pathogen dissemination, could have increased the frequency of specific pathogens, resulting in a skewed distribution of pathogens. A multi-centered study is could reduce such overrepresentation. Neurologic patients are more prone to difficulty swallowing, leading to a higher chance of aspiration pneumonia, which may explain the high proportion of *K. pneumoniae* in the non-ICU setting in our study compared to the study by Chung et al. [8].

## Conclusion

*S. aureus*, *K. pneumoniae*, *A. baumannii*, *P. aeruginosa*, and *E. aerogenes* were the most prevalent pathogens identified in our study. The proportions of each pathogen differed according to whether the patient had an MDR risk factor and whether infection took place in the ICU or not. Resistance to piperacillin-tazobactam and carbapenems in HAP due to Gram-negative pathogens has recently increased, especially in ICU settings.

## Additional file

Additional file 1: **Table S1.** Frequency of MDR or non-MDR isolates in HAP cases with or without MDR risk factors. **Table S2.** Frequency of MDR or non-MDR *S. aureus* isolates in HAP cases with *S. aureus* infection. **Figure S1.** Yearly proportion of isolated pathogens in hospitalized neurologic patients with pneumonia. The ‘Other’ category comprises 30 distinct species. **Figure S2.** The yearly proportion of pneumonia in hospitalized neurologic patients categorized by the location of the infection. **Figure S3.** Antibiotic susceptibility of *S. aureus* in each study period. **Figure S4.** Antibiotic susceptibility of *S. aureus* in each study period. **Figure S5.** Antibiotic susceptibility of *S. pneumoniae* in the early (*n* = 6) and late (*n* = 10) period. (DOCX 538 kb)

## Abbreviations

CAP: Community-acquired pneumonia
CLSI: Clinical & Laboratory Standards Institute
EMR: Electronic medical record
HAP: Hospital-aqcuired pneumonia
HCAP: Healthcare-associated pneumonia
ICU: Intensive care unit
MDR: Multidrug resistance
SNUH: Seoul National University Hospital
