# Supplementary material for: Pneumonia in hospitalized neurologic patients: trends in pathogen distribution and antibiotic susceptibility
Source: Antimicrob Resist Infect Control. 2019 Feb 1;8:25. doi: 10.1186/s13756-019-0475-9 (PMC6359823; doi:10.1186/s13756-019-0475-9)
Supplement: Supplementary file 1 — Table S1. Frequency of MDR or non-MDR isolates in HAP cases with or without MDR risk factors. Table S2. Frequency of MDR or non-MDR S. aureus isolates in HAP cases with S. aureus infection. Figure S1. Yearly proportion of isolated pathogens in hospitalized neurologic patients with pneumonia. The ‘Other’ category comprises 30 distinct species. Figure S2. The yearly proportion of pneumonia in hospitalized neurologic patients categorized by the location of the infection. Figure S3. Antibiotic susceptibility of S. aureus in each study period. Figure S4. Antibiotic susceptibility of S. aureus in each study period. Figure S5. Antibiotic susceptibility of S. pneumoniae in the early (n = 6) and late (n = 10) period. (DOCX 538 kb) [file 13756_2019_475_MOESM1_ESM.docx]

|  | No MDR risk factor | ≥1 MDR risk factor | |
| --- | --- | --- | --- |
|  |  | ICU | Non-ICU |
| MDR | 7 | 58 | 83 |
| Non-MDR | 29 | 19 | 69 |

**Table S1** Frequency of MDR or non-MDR isolates in HAP cases with or without MDR risk factors. Only the top 6 frequent pathogens (i.e. *S. aureus*, *K. pneumoniae*, *A. baumannii*, *P. aeruginosa*, *S. pneumoniae,* and *E. aerogenes*) are counted.

MDR: multi-drug resistance; ICU: intensive care unit.

|  | Early period | | Late period | |
| --- | --- | --- | --- | --- |
|  | ICU | Non-ICU | ICU | Non-ICU |
| MDR | 13 | 14 | 15 | 25 |
| Non-MDR | 2 | 8 | 2 | 11 |

**Table S2** Frequency of MDR or non-MDR *S. aureus* isolates in HAP cases with *S. aureus* infection.

MDR: multi-drug resistance; ICU: intensive care unit.


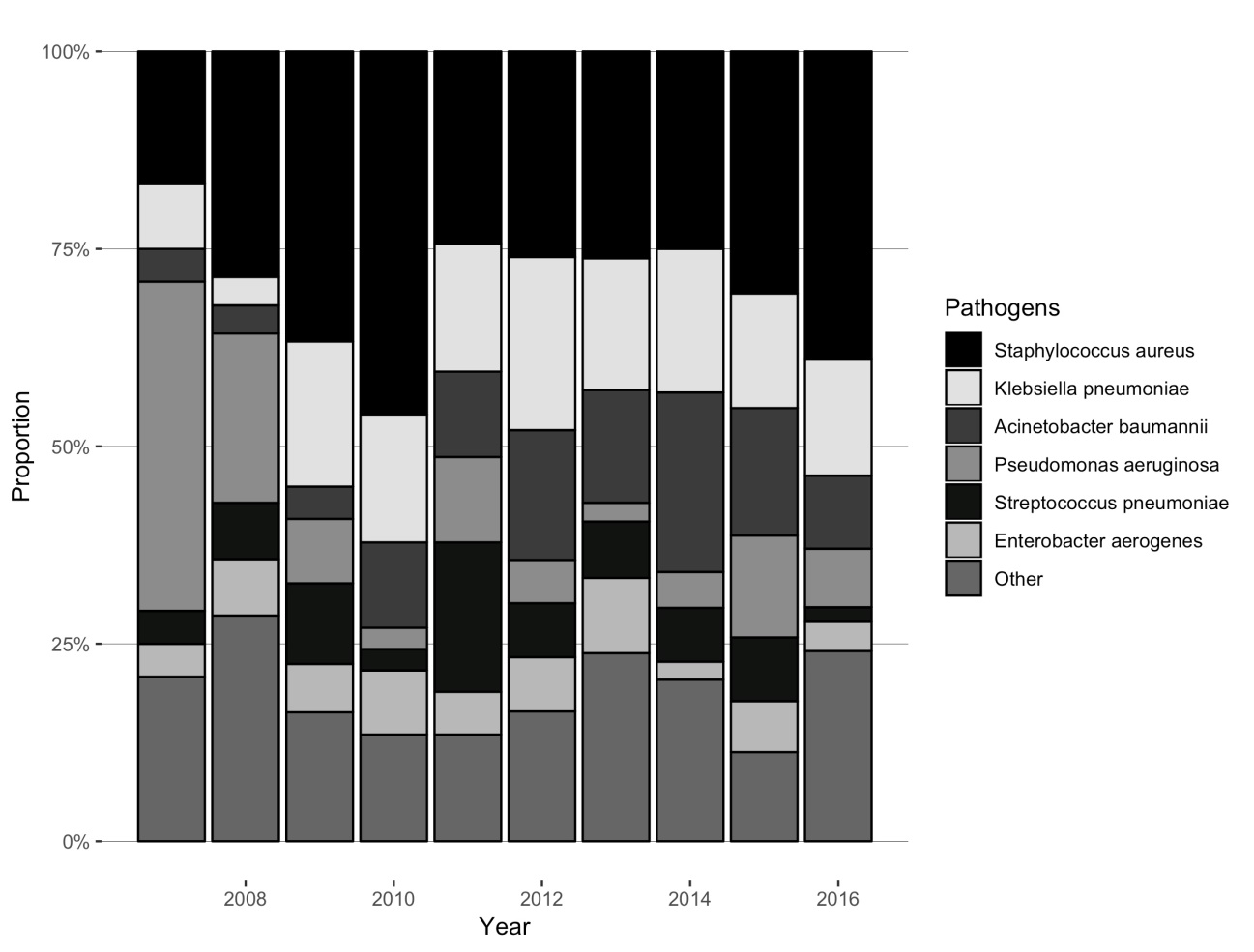


**Figure S1** Yearly proportion of isolated pathogens in hospitalized neurologic patients with pneumonia. The ‘Other’ category comprises 30 distinct species.


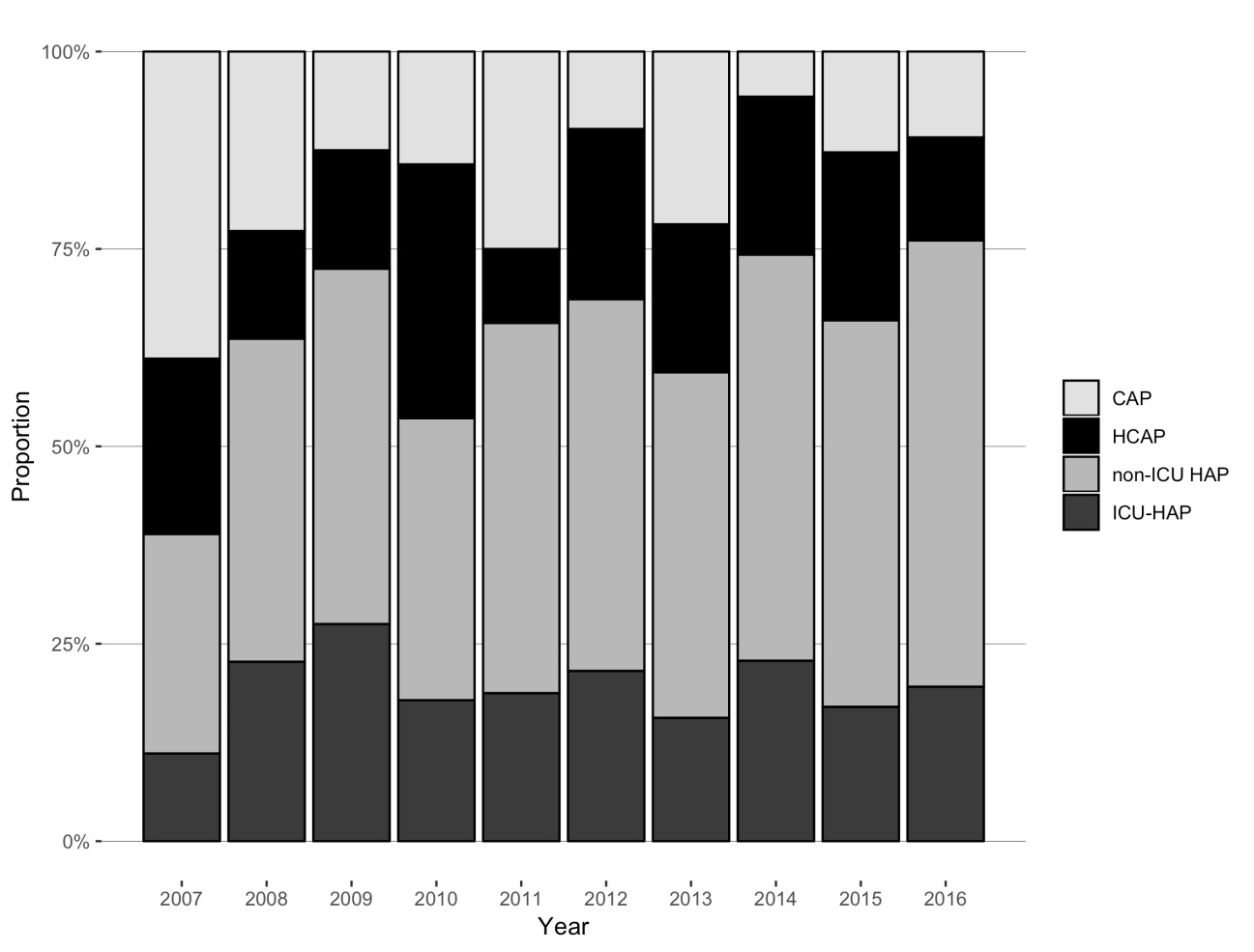


**Figure S2** The yearly proportion of pneumonia in hospitalized neurologic patients categorized by the location of the infection. An overall increasing pattern in the proportion of non-ICU HAP cases is present.


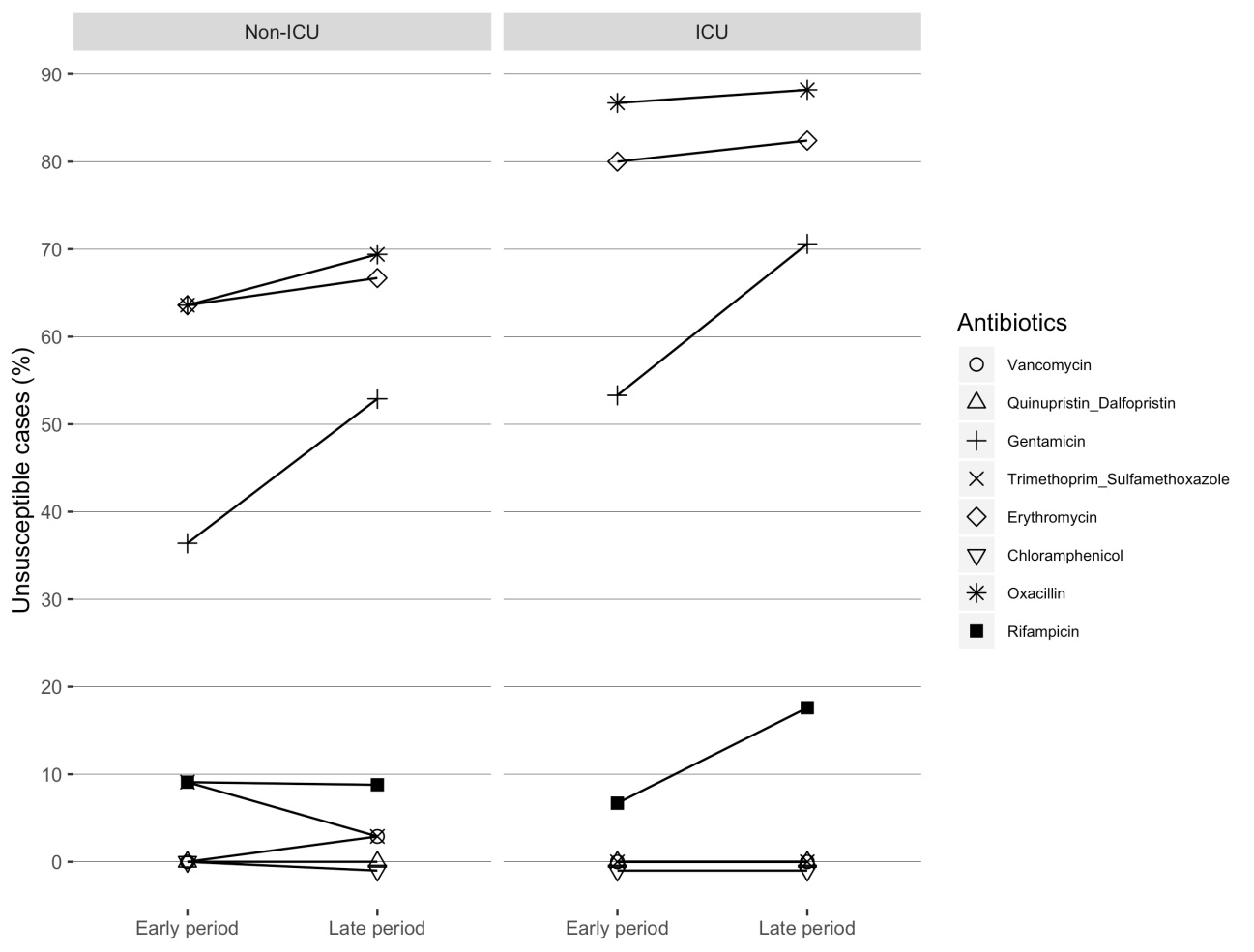


**Figure S3** Antibiotic susceptibility of *S. aureus* in each study period. Additional information of susceptibility to other antibiotic agents are shown in Figure S2.


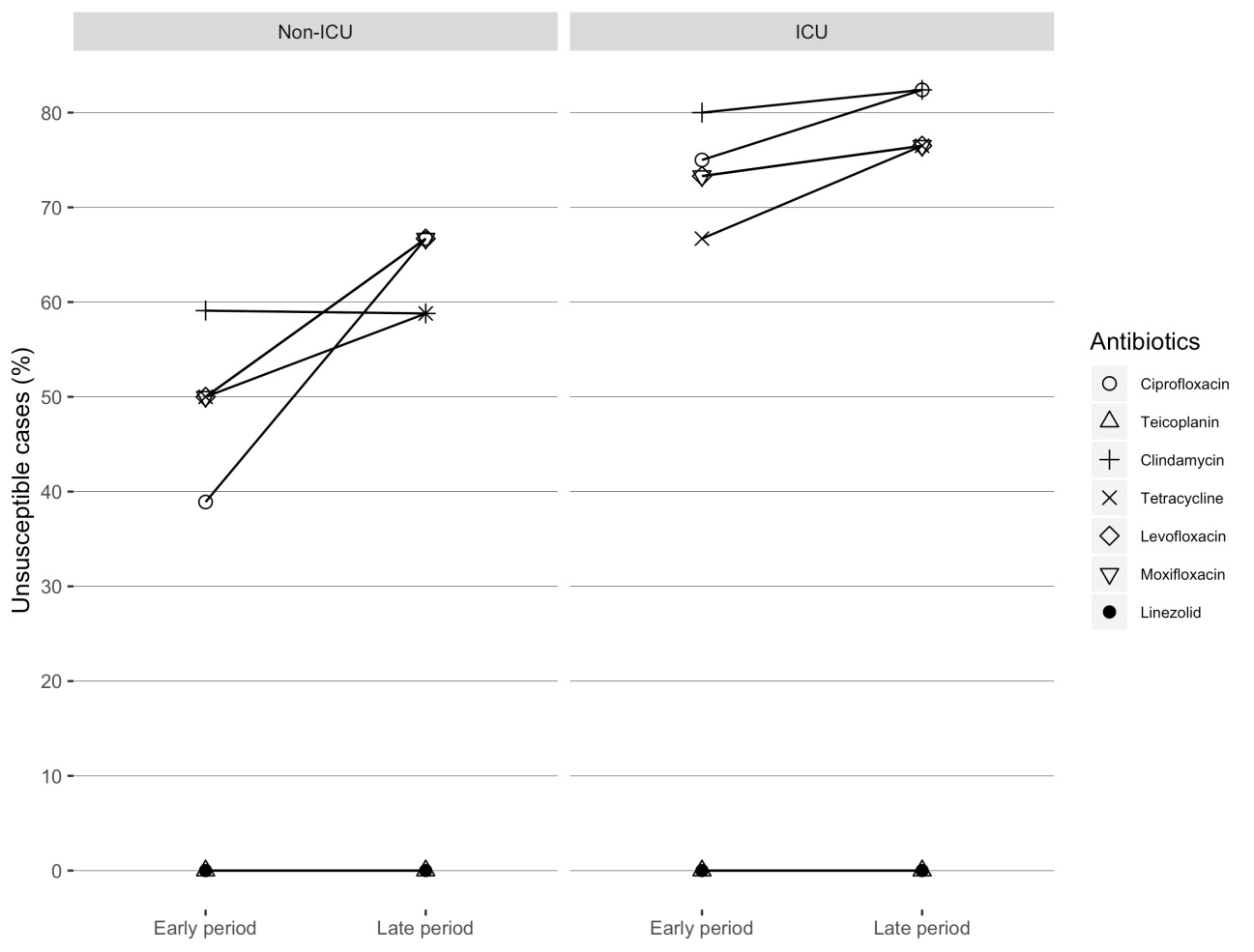


**Figure S4** Antibiotic susceptibility of *S. aureus* in each study period. Continued from Figure S2.


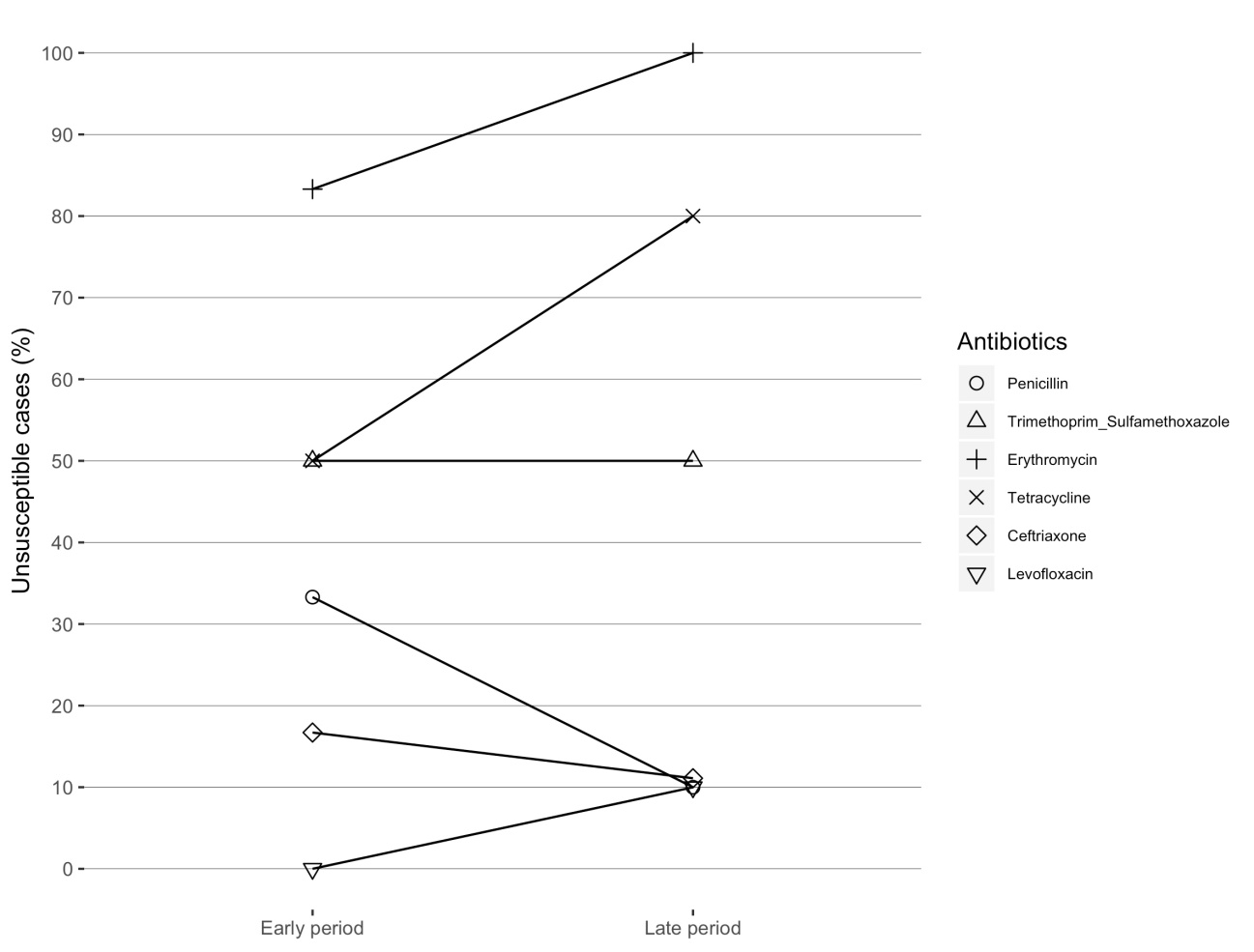


**Figure S5** Antibiotic susceptibility of *S. pneumoniae* in the early (n=6) and late (n=10) period.
